# 경계, 침입, 그리고 배제 : 1946년 콜레라 유행과 조선인 밀항자*

**DOI:** 10.35158/cisspc.2021.10.25.1

**Published:** 2021-10

**Authors:** 정란 김

**Keywords:** 콜레라, 검역, 귀환자, 전후, 미군정, 밀항, cholera, quarantine, repatriates, post-war, SCAP, stowaways

## Abstract

본 논문은 1946년 봄, 귀환선을 통해 중국에서 일본과 38선 이남 해 방조선 (부산항)으로 콜레라가 유입되고 대유행으로 발전하는 과정에 주목한다. 그리고 일본으로 들어간 조선인 밀항자들 사이에서 콜레라 환자와 보균자가 다수 발견된 상황을 연합군 최고사령부 (GHQ/SCAP) 와 일본정부가 어떻게 인식하고 처리해 나가는지에 초점을 맞춘다. 당 시 아시아의 여러 지역에서 콜레라가 유행했는데, 이는 일본의 패전으 로 수 백만 명에 달하는 일본인과 일본의 식민지배하에 놓였던 아시 아인들이 본국으로 귀환하는 과정에서 빚어 진 참사였다. 위의 내용을 검토하기 위해 본 논문에서는 먼저 검역이 포함된 공식귀환작업의 내 용과 성격에 대해 설명한다. 이어 해방 조선과 일본 사이에 ‘밀항’이 라는 비공식적 형태의 이주가 발생하게 된 배경을 추적해 나간다. 나 아가 미군과 일본이 조선과 조선인을 어떻게 콜레라와 같은 감염병과 연결시켜 바라보고, 묘사하고, 그 이미지를 재생산하며 차별과 배제의 논리를 공고히 해 나갔는지에 대한 담론적 관습의 총체에 접근하고자 한다. 이를 통해, 미국이라는 제3자의 개입 하에서 다민족으로 구성되 었던 제국의 과거를 지워버리고, ‘단일 민족국가’로 그 정체성을 재정 립해 나가는 전후 일본사회의 단면을 엿볼 수 있다.

## Abstract (en)

This paper examines the cholera epidemics in Japan and liberated Korea south of 38th degree of latitude; epidemics which appeared to spread from repatriation ships in the spring of 1946. In particular, the paper focuses on how the General Headquarters/Supreme Commander for the Allied Powers (GHQ/SCAP) and Japanese society perceived and handled the situation in which many cholera patients and carriers of cholera bacilli were found among Korean illegal entrants to Japan. At that time, cholera was prevalent in various parts of Asia, which was a disaster facilitated by the repatriation of several millions of Japanese and Japan’s former imperial subjects after the Second World War. First, the paper describes the nature of the repatriation programme including quarantine which was supervised by the US military. Then it examines the reason why illegal shipping developed between liberated Korea and post-war Japan. Finally, the paper will demonstrate how the US military and Japan viewed, imagined and represented Korea and Koreans in connection with infectious diseases such as cholera. This discursive representation was a form of “epidemiological orientalism” which embodied many imperialistic narratives about the oriental origins of diseases such as cholera. Apart from preventing disease, sanitary screening during the process of repatriation was intended to erase Japan’s imperial past and contribute to the rebuilding of Japan as a nation-state under a US-led geopolitical order in East Asia.

## I 이끄는 말

1945년 8월 15일 일본의 패전을 기점으로 전례 없는 규모의 귀환 자,^1)^들이 동아시아 해역을 횡단하며 고국으로 돌아가기 시작했다. 천 황 히로히토 (裕仁)가 연합군에 대해 무조건 항복을 선언할 당시 약 6백90만 명에 달하는 일본 민간인과 군인들이 아시아 태평양 지역에 산재해 있었다. 이에 더해, 일본 내에는 2백 만 명 이상의 조선인과 25,000 명 가량의 대만인 등을 포함해 다수의 피식민지 출신들이 존 재했다. 그 뿐만 아니라 일본의 제국주의 팽창 과정에서 피식민지 간의 이주와 정착도 끊임없이 일어났고, 군인 군속으로 전쟁터에 내 몰린 조선인과 대만인도 적지 않았다. 그 결과 패전 직후 몇 년간 구 (舊) 제국 일본내에서 고국으로 돌아간 귀환자의 총 수는 약 900 만 명에 달했다.^2)^

당시 일본사회에는 빈곤이 만연했고, 국민들은 과도한 피로와 패 전의 절망감으로 인해 ‘허탈상태’에 빠져 있었다.^3)^ 오랜 세월 일제의 인적 물적 수탈의 대상이 되었던 조선을 비롯해 일본의 피식민지와 점령지는 그 사정이 더욱 심각했다. 설상가상으로 전쟁으로 인한 물 자 부족과 의료보건체계의 붕괴로 인해 여러 감염병이 창궐했고, 대 규모 귀환작업이 진행되는 과정에서 각지로 퍼져 나갔다. 패전으로 인해 황폐해진 일본은 물론 식민통치에서 해방됨과 동시에 남북으로 분단 된 조선에 있어서 감염병의 유행은 사회적 혼란을 가중시키는 요인이 되었다. 뿐만 아니라 38선 이남의 조선과 일본을 통치하게 된 미군 역시 주둔군의 건강을 위협하고 점령지 내의 안정을 해치는 감염병의 유행에 신경을 곤두세웠다. 이에 연합군최고사령부 (General Headquarters/Supreme Commander for the Allied Powers, 이하 SCAP)는 귀환자 검역을 통해 감염병의 전파를 차단하고 권역 내의 인적 물적 흐름의 통제를 철저히 해 나가고자 했다. 감염병 중에서 특히 우려 되었던 것은 콜레라 (cholera)^4)^였다. 콜레라는 1946년 봄 중국에서 들 어온 귀환선을 통해 일본과 38선이남 조선으로 유입되었다. 이 시기 일본으로 밀항해 가는 조선인들이 늘어나기 시작했는데, 이들은 일 본으로 콜레라가 전파되는 원인으로 지목되었다. 또한 일본사회는 조선인 밀항자를 공중보건과 치안문제로 연결시키며 재일조선인 사 회에 대한 제재를 강화하는 논리로 발전시켰다.

최근 들어 구 일본제국 내의 귀환작업과 재일조선인 사회에 대한 연구가 다각적으로 진행되고 있다. 그 중, 전후 일본이 어떠한 경위 로 재일조선인에 대한 차별과 배제의 논리를 구체화해 나갔는지에 대해 천착한 조경희의 논문 “전후 일본 ‘대중’의 안과 밖” (2013)은 주목할 만하다. 조경희는 해당 논문에서 SCAP이 부여한 모호한 법 적지위로 인해 재일조선인들이 ‘제3국인’이라 불리우게 되고 밀항과 암시장 등 불법행위의 주체로 묘사되면서 단속의 대상이 된 배경을 자세히 밝히고 있다.^5)^ 이와 관련해서 최덕효의 논문 “The Empire Strikes Back from Within” (2021) 역시 시사하는 바가 크다. 그는 해 방민족 (liberated people) 이면서 동시에 비 (非)일본계 일본국적자인 재일 조선인들을 두고, 패전 직후 일본사회가 그들의 범법행위로 인 한 사회적 무질서를 부각시키며 재일조선인에 대한 차별을 정치담론 화 해 가는 과정에 초점을 맞춘다. 최덕효는 일본 사회의 이러한 움 직임은 약화된 경찰권의 회복을 꾀하면서 민주주의적 개혁을 추진하 는 미군의 점령통치에 대한 우회적인 비판을 목적으로 한다는 점도 지적한다.^6)^ 테사 모리스-스즈키 (Tessa Morris-Suzuki) 는 전후 일본의 국경 통제와 재일조선인 문제를 좀 더 심층적으로 분석하고 있다. 그녀는 저서 “Borderline Japan” (2010) 에서 일본의 현재 재일조선인/한국인의 법적 지위문제가 전후 귀환자 관리에서부터 시 작되었고, 다민족으로 구성된 제국에서 ‘단일민족국가’로 일본이 재 편되어 가는 과정과 그 궤를 같이 하고 있다는 점에 주목한다.^7)^

이들 세 연구는 공통적으로 조선인 밀항자들 사이에서 발생한 콜 레라가 전후 일본사회가 재일조선인들에 대한 차별과 배제를 강화해 나가는 근거로 작용되었다는 점을 지적한다. 그러나 콜레라의 유행 은 하나의 사례로 포함되었을 뿐, 유행의 역사적 경위나 감염병을 둘러 싼 사회적 인식에 대한 고찰은 주요 논지에서 벗어나 있다. 그 로 인해 일본사회가 어떻게 콜레라 유행과 조선(인)을 유기적으로 연결시키며 경계와 배제의 논리를 강화했는지에 대한 설명이 결여되 어 있다. 이에 대한 논의를 심화하기 위해서는 당시 일본 사회에 뿌 리 깊게 자리한 아시아에 대한 병리학적 심상지리의 기원과 그 발현 에 대해 살펴볼 필요가 있다. 이와 관련해 김정란은 “제국의 흔적 지 우기” (2021)에서, 아시아를 ‘질병의 온상’으로 바라보던 미국과 일본 의 시선이 귀환자 검역에서도 드러났다는 점을 밝히고 있다. 그러나 해당 연구는 전후 일본에서 실시된 귀환자 검역과 감염병의 통제에 초점을 맞추고 있기 때문에 밀항 조선인들과 콜레라에 대한 구체적 인 설명은 생략되어 있다.^8)^

위에서 언급한 선행연구의 성과와 문제의식을 바탕으로, 본 논문 에서는 구 일본제국 내에서 귀환작업이 한 창이던 1946년 봄에 일본 과 38도선 이남 조선에 유입된 콜레라의 유행상황에 대해 살펴본다. 이를 위해 우선 검역을 포함한 공식 귀환작업의 내용과 목적에 대해 설명하고, 해방 조선과 일본사이에 밀항이라는 비공식적 형태의 이 주가 발생하게 된 배경도 함께 추적한다. 이어 밀항선을 타고 일본 으로 들어간 조선인들 사이에서 콜레라 환자와 보균자가 다수 발견 된 상황을 SCAP과 일본정부가 어떻게 인식하고 처리해 나가는지에 대해 고찰한다. 나아가 미군과 일본이 조선과 조선인을 어떻게 콜레 라와 같은 감염병과 연결시켜 바라보고, 묘사하고, 그 이미지를 재생 산하며 차별과 배제의 논리를 공고히 해 나갔는지에 대한 담론적 관 습의 총체에 접근하고자 한다.

## II 귀환자 검역

일본은 제2차 세계대전에서 패하면서 제국주의의 막을 내렸고, 동 아시아의 지정학적 역학구도는 미국과 소련이 대립하는 형태로 재편 되어 나갔다. 그리고 패전국 일본과 38선 이남의 해방조선은 미군의 통치 하에 놓이게 되었다. 1945년 9월 2일, 일본으로부터 정식으로 항복조인을 받은 미육군태평양사령부 사령관 맥아더 장군은 연합군 최고사령부 (SCAP)를 도쿄에 설치하였다. 같은 달 7일, 조선의 북위 38도선 이남에도 재조선미육군사령부군정청 (United States Military Government in Korea)이 설치되면서 미군정이 시작되었다. 재조선미 군사령관에 임명된 미 육군 제24군단장 하지 중장 (John R. Hodge)은 8일 인천에 도착했고, 이튿날 총독부 건물에서 조선총독 아베 노부유 키 (阿部信行)로부터 정식 항복선언을 받았다. 이로써 1948년 8월 15 일 대한민국 정부가 수립될 때까지 미군정 통치가 이어지게 되었다. 이처럼 일본제국의 해체는 미군이 주축이 된 연합군이라는 강력한 제3자의 개입 (“The Third-Party-Decolonization”)으로 빠르게 진행되어 갔다. 그로 인해 조선과 일본 사이의 탈식민지화는 물론 일본의 전쟁 책임에 대한 논의가 제대로 이루어지지 못하는 결과를 초래했다.9)

38도선 이남 조선에서는 9월 28일부터 미군 제40보병사단의 감독 하에 미군 제160보병연대가 담당하는 공식적인 귀환작업이 개시되었 다. 일본과 지리적으로 인접하고 조선 내의 다른 항구에 비해 항만 과 여타 제반 시설이 잘 갖춰진 부산항이 주요 귀환항으로 정해졌 다.^10)^ 부산은 조선중기부터 왜관 (倭館)을 중심으로 조·일간 외교와 무역의 거점이 되었다. 그리고 개항기부터 식민지기에 걸쳐서는 일 본과 아시아대륙을 잇는 연결점으로 설정되었다.^11)^ 미군에 의한 공 식 귀환작업이 시작되면서, 재조일본인들과 무장해제 된 일본군 대 부분은 부산항 1번 부두에서 일련의 절차를 거친 후 귀환선에 몸을 실었다. 일본에서 돌아오는 대다수의 조선인들 역시 부산항을 통해 귀환절차를 마친 후 목적지로 향했다.^12)^

부산항에서 실시된 귀환작업 중에서 무엇보다 중요한 절차는 입출 항 검역이었다. 전례 없는 규모의 귀환자들이 이동하는 과정에서 적 절한 예방 조치가 취해지지 않는다면 각종 질병이 전파될 수 있다는 점을 미군은 일찍부터 인지했다.^13)^ 일본과 남한에서의 군정통치를 준비하는 과정에서 미군이 무엇 보다 우려했던 점은 낯선 자연환경 과 각종 질병의 유행이었다. 미국은 다른 서양열강과 마찬가지로 아 부산항에서 실시된 귀환작업 중에서 무엇보다 중요한 절차는 입출 항 검역이었다. 전례 없는 규모의 귀환자들이 이동하는 과정에서 적 절한 예방 조치가 취해지지 않는다면 각종 질병이 전파될 수 있다는 점을 미군은 일찍부터 인지했다.^13)^ 일본과 남한에서의 군정통치를 준비하는 과정에서 미군이 무엇 보다 우려했던 점은 낯선 자연환경 과 각종 질병의 유행이었다. 미국은 다른 서양열강과 마찬가지로 아

미군은 일본과 남한을 비롯한 점령통치지역에서의 감염병 관리를 위해 의료관련 정보 보고서를 활용했는데, “육해군 합동정보연구 (Joint Army-Navy Intelligence Study)”가 그 대표적인 예라 할 수 있다. 제2차 세계대전이 막바지로 이를 무렵, 미군은 일본의 패전을 상정 하고 일본과 그 식민지에서의 군사작전과 점령을 준비하기 위해 “육 해군 합동정보연구”라는 타이틀의 연속 보고서를 작성했다.^15)^ 해당 보고서에는 “건강과 위생 (Health and Sanitation)” 섹션이 포함되었는 데, 해당 지역의 전반적인 의료시스템과 위생환경은 물론 미군이 특 별히 조심해야 할 감염병 등이 자세히 기술되어 있다. 일본의 경우, 발진티푸스, 디프테리아, 천연두, 이질, 그리고 식중독에 의한 설사병 등이 만연한다고 보고되었다. 또한 군사작전에 잠재적인 위험이 될 수 있는 감염병으로 말라리아와 콜레라가 포함되었다.^16)^ 식민지 조 선의 경우 열악한 위생환경과 부족한 의료시설로 인해 사람들이 여 러 감염병의 위험에 상시 노출되어 있다고 설명했다. 이는 조선총독 부가 “일본인의 주도하에, 일본인이 강제하는 형태로, 일본인이 중심 이 된” 보건위생 행정을 펼쳤기 때문이라고 지적하고 있다. 그리고 미군이 특히 조심해야 할 질병으로 말라리아, 이질, 성병, 동상 등을 꼽았다.^17)^ 이처럼 다양한 질병의 만연과 전파를 우려했던 미군에게 있어 “청결습관이 없는 귀환자들”이 범람하는 입출항에서의 위생관 리와 감염병 통제는 만전을 기해야 하는 문제였다.^18)^ 더욱이 38도선 을 사이에 두고 소련과 대치하는 상황에서 통치지역의 안정은 미군 에게 있어 무엇보다 중요했는데, 물자부족에 허덕이고 의료체계가 붕괴된 일본과 조선에 감염병이 전파된다면 사회적 혼란이 더욱 가 중될 것으로 예상되었다.^19)^

부산항에서 실시된 귀환자 검역과 입출국절차를 살펴보면 다음과 같다. 일본인 귀환자들의 경우, 우선 부산항에 설치된 집결지역 (Assembly Area)에서 대기하다 차례가 되면 DDT를 이용한 이 박멸 작업을 거친다. 그 다음 천연두와 발진티푸스, 장티푸스, 파라티푸스 등의 감염병에 대한 예방접종을 마친다.^20)^ 검역절차가 끝나면 귀환 자들은 외환교환소에서 환전을 한다 (그림 1). 조선에서 축적한 부 (富)는 조선에 귀속되어야 한다는 원칙 하에서, 일본인 한 사람당 본 국으로 가지고 갈 수 있는 재화는 현금 1,000엔과 가방 하나로 한정 되었다. 따라서 검역절차를 마친 귀환자들이 귀환선에 승선하기 직 전 미군은 그들의 몸과 가방을 수색해서 밀수품이나 제한된 금액 이 상의 현금과 귀금속이 없는지 살폈다 (사진 1).^21)^ 부산항으로 들어온 조선인 귀환자들의 경우, 미군 의무과와 조선인 구호 단체들의 지시 에 따라 하선 후 100명씩 무리를 지어 1번 부두를 빠져 나와 수용소 로 향했다. 그 곳에서 DDT로 소독을 한 후 환전소에서 가져온 엔화 를 교환했다.^22)^

한 편, 일본에서의 귀환자검역은 부산항에서 보다 조금 늦게 시작 되었다. 1945년 9월 SCAP은 일본정부를 대상으로 각서 “공중보건위 생에 대한 건 (SCAPIN-48)”를 발표하였다. 총 9항으로 구성된 이 각 서는 일본 내 위생환경 개선과 질병관리에 관한 지시를 담고 있다. 그 중 제6항은 “미 해군과 협력하여 해항검역을 실시할 것. 해항검 역은 군 (軍)이 아니라 문민통제 (civil control)로 착수할 것”을 지시 하고 있다.^23)^ 이를 기점으로 일본의 주요 항에 귀환자 수용소와 검 역소가 설치되기 시작했다. 그리고 같은 달 24일, 후생성 분과규정을 개정해서 위생국에 임시검역과를 설치하였다. 약 한 달 후인 1945년 10월 27일, 다시 임시방역국을 설치하여 그 안에 검역과를 두었다. 그 보다 일주일 앞선 10월 20일에는 “귀환에 관한 상륙 및 항만위생 에 대한 의학, 위생학적 조치에 관한 건”이라는 제목의 각서가 발표 되었다. 상기의 제반 과정을 거친 끝에, 10월 중순부터 우라가 (浦 賀), 센자키 (仙崎), 하카타 (博多), 사세보 (佐世保), 가고시마 (鹿児 島)항을 필두로 귀환자 검역이 부분적으로 시작되었다. SCAP의 명 령에 따라 귀환업무의 일원화를 위해 “지방인양원호국관제 (地方引 揚援護局官制)”가 11월 24일에 공포되었고, 3일 후에 지방인양원호국 이 설치되었다. 이 즈음 11개의 귀환항이 운영되고 있었는데, 매일 약 45,500 명의 일본인 귀환자를 수용할 수 있는 규모였다.^24)^ 12월에 들어 모든 귀환항에서의 검역이 공식적으로 실시되기 시작했다.^25)^ 일본에서 실시된 입항검역의 내용을 살펴보면 다음과 같다. 우선 귀환선은 항구의 근해에 닻을 내려 대기하고, 검역관들이 승선을 하 여 출발항에서 발급한 증명서 확인과 함께 감염병 환자의 유무를 살 핀다. 감염병의 잠복기를 고려해 출발항에서 승선한 날짜까지 소급 하여 최소 6일의 기간 동안 선내에 감염병 환자가 없는 것으로 확인 되면 입항을 허락한다. 만약 선내에 감염병 환자나 보균자가 발견되 면 이들을 격리병원으로 즉시 옮기고, 나머지 승객들은 추가 발병의 유무를 살피기 위해 14일간 선내에 머물러야 한다. 이후 추가로 발 병하는 자가 없으면 입항이 허락되고, 만약 추가로 발병한 자가 나 오면 환자는 격리병원으로 옮겨지고 나머지는 마지막 발병자가 나온 날로부터 추가로 14일 간 선내에서 대기해야 한다.^26)^ 하선이 허락된 귀환자들이 배에서 내리면 검역관은 DDT를 살포하면서 그들의 몸과 소지품을 소독한다. 이 과정은 마치 그들의 몸에 새겨 진 ‘제국의 흔 적’을 씻어내는 듯한 행위이자 미군에 의해 ‘불결하고 병든 신체’가 정화 (淨化)’된다는 시각적 심리적 효과도 내포했다.^27)^ 이후 귀환자 들은 약욕 목욕을 하고 천연두와 장티푸스에 대한 예방접종을 받았 다. 모든 귀환 절차가 끝나면 귀환자들은 수용소를 떠나 목적지로 향할 수 있었다.^28)^

## III 귀환선이 싣고 온 콜레라

일본의 귀환항에서 실시된 검역은 부산에서 진행되는 것 보다 더 철저하게 이루어졌는데, 귀환자 체내의 병원체 유무를 확인하는 정 밀검사도 포함되었다. 귀환자 검역의 주된 목적이 ‘질병의 온상’이라 여겨지는 아시아 태평양 각지에서 돌아온 이들로 인해 감염병이 유 입되는 것을 차단하는 것으로, 감염병 환자를 격리하는 것만큼 보균 자를 발견하는 것도 매우 중요했다.

미군과 마찬가지로 일본은 아시아를 ‘미개’한 지역으로 보았고, 감 염병이 창궐하는 원인을 그들의 ‘야만적 습관’때문이라고 강조해 왔 다. 이러한 태도는 일본이 메이지 유신을 단행하며 서구 열강과 동 등한 위치에 서기 위해 국내의 근대화와 제국주의팽창을 동시에 시 작하면서부터 나타났다. 인종적으로 매우 유사한 아시아국가들과 자 국을 구분하기 위해 일본은 그들의 기준으로 ‘문명화 정도 (程度)’의 차이를 강조했다. 나아가 제국주의 팽창과정에서 일본은 아시아에 대한 ‘문명화의 사명’을 내세웠는데, 근대화 과정에서 발빠르게 도입 한 서양의학이 그 대표적인 수단 (tool)으로 채택되었다. 서구열강은 선진의학기술과 공중보건시스템을 바탕으로 감염병을 관리해 나갔 고, 이를 인종적 우월성과 연결시키며 제국주의 침략의 정당성을 획 득하려고 했다.^29)^ 일본은 이러한 서구열강을 모방하는 형태로 제국 주의 팽창을 기도한 것이다. 이러한 움직임은 메이지 유신을 단행하 고 10 년이 채 되지 않아 부산을 개항하고 난 직후부터 나타나기 시 작했다. 1876년 조일수호조규가 체결된 직후, 일본 관리와 의사 한 명이 서울로 향하기 위해 부산으로 들어왔다. 부산에 머무는 동안 일본인 의사는 조선인 환자들을 치료했는데 이는 조선인들을 회유하 고 일본의 ‘선진문명’을 보여주기 위한 목적이라고 설명했다.^30)^ 이처 럼 근대 일본은 조선을 포함한 주변 아시아를 미개하고 질병으로 가 득 찬 곳으로 바라보았는데, 이러한 부정적인 인식은 그곳을 떠나 본국으로 돌아오는 귀환자들에게도 투영되었다. 그리고 이들을 잠정 적 보균자로 간주하며 검역을 통해 체내에 있는 병원성 요소를 걸러 내고자 했다.^31)^

검역관은 귀환자들에게서 채취한 혈액과 대변 검체를 검사하며 말 라리아나 장티푸스, 이질과 같은 질병의 감염 여부를 살폈다.^32)^ 병원 체 유무를 확인하는 정밀검사는 1946년 봄 콜레라가 귀환선을 통해 유입되면서 더욱 중요하게 다뤄졌다. 1946년 3월 29일 중국 광동 (廣 東)을 떠나 일본 우라가항으로 향하던 귀환선에서 콜레라 의심환자 가 발생했다. 출항할 당시에는 콜레라 의심환자는 발견되지 않았지 만, 광동항을 떠난 지 3일째 되는 날부터 콜레라 증상을 보이는 이 들이 발생하기 시작했다.^33)^ 이 배는 4월 5일 우라가에 입항했는데, 배 안에는 콜레라 증상으로 사망한 사체 3 구와 콜레라 의심환자 17 명이 타고 있었다. 이후 광동, 아모이 (샤먼, 廈門) 및 하노이를 출발 해 우라가로 향한 귀환선 사이에서 계속해서 콜레라 환자와 의심자 가 발생했다. 5월4일 조사에 따르면 그 때까지 우라가로 들어온 귀 환선에서 총 1,593명의 환자와 1,921명의 보균자, 그리고 총 169명의 선내 사망자가 보고되었다.

콜레라는 1945년 11월경부터 인도와 중국에서 유행하기 시작했다. 당시 미군은 수 백만명에 달하는 전쟁난민과 조선인, 일본인 등이 본국으로 귀환하는 과정에서 미군의 관할지역으로 콜레라가 전파될 가능성을 우려했다. 따라서 미군의 통치하에 놓인 지역에서 장 관련 질환 (enteric diseases)이 발생할 때마다 콜레라의 가능성을 고려해 엄중히 대처하도록 권고하였다.^34)^ 그러나 연합군최고사령부의 보건 복지과장으로 역임한 크로포드 샘스 (Crawford F. Sams) 준장의 회고 록에 따르면, 광동에서 우라가로 콜레라가 전파된 것은 당시 중국에 주둔하고 있던 미군의관이 출항 검역절차를 제대로 관리하지 못한 것에서 기인했다.^35)^ 이후 일본의 다른 귀환항에도 콜레라를 실은 귀환선이 입항했는데, 5월에는 방콕에서 사세보항 그리고 상해에서 하카타항으로, 6월에는 군산과 후루다오 (胡盧島, Huludao)에서 하카 타항(博多)으로, 7월에는 옛 만주지역과 조선에서 마이즈루항 (舞鶴) 으로 들어온 귀환선에서 콜레라가 발견되었다.^36)^ 1946년 12월까지 일본 귀환항에서 검역을 받은 선박 수는 총 4,700 척이고 이 중 일 본인 귀환자를 태우고 온 것은 3,632척이었다. 병원체 검사 총 수는 2,427,649 건으로, 그 중에서 콜레라 양성이 2,259 건 보고되었다.^37)^ 귀환항에서는 귀환자를 대상으로 한 예방접종도 철저히 실시했지만, 콜레라는 결국 일본국내로 전파되어 총 1,245명의 환자가 발생했 다.^38)^

콜레라가 귀환선을 통해 일본으로 유입되자, 38선 이남 조선에 주 둔하던 미군도 콜레라 검역을 강화했다. 미군정의 군무국장 찰스 에 니스 대령 (Colonel Charles Ennis)은 4월 13일 “귀환자들의 콜레라 검 역 절차 (Quarantine Procedures for Cholera in Repatriates)” 라는 제목 의 문건을 통해 중국을 출발해 귀환항으로 들어오는 선박에 대해 콜 레라 검역을 철저히 실시할 것을 지시했다. 그 일환으로 중국에서 들어오는 귀환선을 대상으로 콜레라 (의심)환자의 유무에 상관없이 입항 전에 의무관이 승선해서 승객들의 상태를 철저히 살필 것을 명 령했다. 콜레라 환자가 선내에 있을 경우, 항만 인근에 미군이 승인 한 엄격한 격리 시스템을 갖춘 병원이 있을 경우에 한해서 환자를 그곳으로 옮길 것을 지시했다. 만약 적절한 격리병원이 마련되어 있 지 않다면 환자를 선내에서 격리시킬 것을 명했다.^39)^

일본에 콜레라가 전파되고 약 한달 후, 부산항에도 콜레라가 유입 되었다. 중국 광동에서 조선인 귀환자 3,100 여 명을 태우고 5월 1일 부산으로 들어온 귀환선 내에 콜레라 환자가 발생한 것이다. 익일 콜레라 환자는 선내에서 사망했고, 그 후로도 의심환자가 발생했다. 이 귀환선에는 전쟁 중 보국대로 끌려갔다 돌아온 홍문화라는 이름 의 의사가 타고 있었는데, 선내의 참혹한 상황을 설명하며 “배 안은 협착하고 불결하야 방역이 아니되니 상륙을 시켜 완전이 방역이 될 때까지 격리를 시켜주든지, 부산에 적당한 시설과 장소 없거든 방역 설비가 있는 다른 항구로 돌려 상륙을 시켜달라” 고 탄원했다. 홍문 화의 탄원서에 따르면 미군은 콜레라로 사망한 자의 시체만 가져갔 을 뿐, 격리시설이 불충분하다는 이유로 환자를 선내에서 격리시키 고 일반승객도 배 안에 머무를 것을 명령했다.^40)^

일본에서도 미군이 발표한 ‘해상인양선내정류격리법 (海上引揚船 內停留隔離法)’에 따라 건강한 승객들은 마지막 환자가 보고되고 2 주가 지날 때까지 좁은 선내에 머물러야 했다. 그러나 선내의 위생 환경은 열악했는데 특히 화장실은 소독법을 실시하기에 적당한 형태 가 아니었다. 또한 승객들은 식음료와 생활용수 부족에 시달려야만 했다. 이처럼 승객들은 열악한 환경에 노출된 채 음식물 섭취도 제 대로 하지 못했는데, 그 결과 콜레라환자가 선내에 추가로 발생하는 경우도 생겨났다. 그러나 부산과 달리 콜레라 환자들은 즉시 항만 부근의 격리병원으로 이송되었다.^41)^ 부산항의 경우 격리병원이 제대 로 갖춰져 있지 못해 콜레라 환자를 한동안 선내에서 격리를 하는 상황이라, 다른 승객들에게 콜레라가 전파될 위험이 더욱 높았다. 뿐 만 아니라 그 배안 승객들 중에는 파라티푸스와 말라리아 환자도 상 당수 존재했지만 그들 역시 한동안 하선이 허락되지 않았다.^42)^ 5월8 일이 되어서야 경남도청은 선내 환자를 신선대 검역소에 수용하기로 결정했다. 같은 선내의 일반 승객에 대해서도 해당 검역소에서 대소 변 검사를 실시하여 음성결과가 나오면 상륙시키기로 했다.^43)^

설상가상으로 미군의 (美軍醫)의 오판까지 더해져 부산항에서는 국내 유입을 막기 위한 콜레라 방역이 지체되었다. 5월 7일을 기점 으로 해당 선박에서는 12명의 콜레라환자와 11명의 사망자가 발생한 상태였다. 경상남도청 보건후생과에서는 국내로 콜레라가 전파되는 것을 막기 위해 중앙군정청에서 콜레라 예방주사를 받아와서 필수인 력을 대상으로 접종할 계획을 세웠다.^44)^ 그러나 미군정청 인사는5월 중순 정례기자회견에서 콜레라 환자가 발생한 상기의 귀환선을 미군 의가 조사중인데, 콜레라균은 아직 발견되지 않았다고 설명했다. 또 한 선 내에 콜레라와 같은 설사병은 있지만, 만약 그것이 콜레라라 면 사망자가 속출할 것인데 그렇지 않으므로 감염병이 아닌 것은 사 실이라고 덧붙였다. 보건후생과에서 진성 (眞性) 콜레라라고 발표한 것에 대한 질문에는 “그것은 조선인 의사와 미군의사의 의견이 갈린 것”이라고 일축했다.^45)^ 이러한 입장은, 상기한 바와 같이 1945년 11월 콜레라가 중국과 인도에서 유행한다는 보고에 따라 점령지역 내에서 장 관련 질환이 발생할 시 콜레라의 가능성을 고려해 대처하도록 한 미군정청의 권고에도 위배된다. 결국 20일 경 부산시 내에서 콜레라 의심환자가 네 명이 발생했고, 그들은 모두 당일 사망했다. 사망자 인근에 10 여명의 동일증상의 환자가 연이어 발생하여, 시 방역과는 21일 경찰의 협력 하에서 환자 발생지역 주변의 교통을 차단하고 소 독을 대대적으로 실시했다.^46)^ 5월 23일자 부산신문의 “영도필담”에 실린 글은 미군의의 오판에 대해 다음과 같이 풍자하고 있다.^47)^

“호열자란 배가 부산항에 정박중이라고 방역 [의용대] 전시편성까지 하드니 적은 상륙한 모양인가. 저번 귀환선 조사결과 미군의는 호열자 균은 발견 못했다고 하고 조선의사는 호열자라고 판단. 균이 동양 (중 국)이라 조선의사 판단이 옳았든가? 어쨌든 전장 (戰場)은 조선이니 조 선의사만 믿소.”

5월 중하순부터 콜레라는 부산시내로 급속도로 퍼졌고, 곧이어 다 른 지역에서도 콜레라 환자가 속출하기 시작했다. 5월 22일을 기점 으로 미군정청 보건후생부에 보고된 바에 따르면, 부산에 40명, 대전 에 3명, 인천에 1명의 콜레라 환자가 발생했다. 대전에서 발생한 환 자들은 상해에서 부산항으로 돌아온 징병군인 출신으로 고향인 전북 으로 향하던 중 대전에서 발병한 것이다. 인천의 환자 역시 상해에 서 부산으로 들어와 대전, 서울, 개성을 거쳐 인천에 도착해 19일 발 병했다.^48)^ 이렇게 콜레라가 귀환선을 통해 부산으로 유입되고 전국 으로 확산되어 나가는 상황을 맞아 미군정과 보건당국은 부산항에서 의 검역을 한층 강화해 나갔다. 우선 격리병실 등이 완비된 옛 일본 병원선 1척을 귀환자를 위한 병원선으로 사용하기로 결정했다. 뿐만 아니라 감염병 예방책으로 귀환선박의 검역과 예방주사접종을 매일 시행하기로 했다.^49)^

그러나 콜레라는 점차 다른 지역으로 확산되었고, 6월 21일 기준 으로 38도선 이남의 48개 지역에서 환자 1,335명과 사망자 592명을 기록했다.^50)^ 7월에 들어서면서 38선 이북에도 콜레라가 유행하기 시 작했는데, 평양, 원산 그리고 평양으로 향하는 철도선 주변지역에서 콜레라 환자가 보고되었다.^51)^ 8월에는 중국에서 이북으로 돌아온 군 인들 사이에서 콜레라가 발생했다. 열악한 위생환경으로 인해 콜레 라는 해방조선에서 빠른 속도로 퍼져 나갔고, 당해 38선 이남에서만 총 17,000명의 환자와 11,000 명에 이르는 사망자를 기록했다.^52)^

## IV ‘외부로부터의 위협’: 조선인 밀항자들과 콜레라

이처럼 콜레라는 귀환선을 타고 일본과 해방조선 이남으로 유입되 었고, 두 지역에서 모두 국내 유행으로 이어졌다. 위에서 살펴본 것 처럼, 6월과 7월에는 38도선 이남에서 일본으로 들어간 선박에서도 콜레라가 발견되었다. 그런데 이 무렵부터 일본으로 들어오는 조선 인 밀항자 문제가 점차 부각되었는데, 설상가상으로 밀항자들 사이 에서 콜레라 환자와 보균자가 다수 발견되었다. 그 결과 일본사회와 SCAP은 이들을 일본으로 콜레라가 유입되는 주요원인처럼 다루기 시작했다.

밀항선을 타고 대한해협을 건너는 사람들은 미군이 부산항에서 귀 환자 관리를 공식적으로 개시하면서부터 나타나기 시작했다. 일본인 들의 경우 무장해재 시킨 군인들을 우선해서 본국으로 돌려보냈기 때문에, 민간인들은 귀환선에 오르기까지 약 3주에서 한 달 가량 부 산항에서 대기해야 했다. 일본인 귀환자들이 머무를 수 있는 임시숙 소가 부산항 부근에 마련되었지만, 귀환선을 타기 위해 밀려드는 이 들을 전부 수용하기에는 턱없이 부족한 공간이었다. 따라서 많은 이 들이 화물차나 기차역 플랫폼에서 잠을 청해야 했다.^53)^ 뿐만 아니라, 부산항에서 실시된 귀환자 검역은 그 절차가 까다로웠고 1인 당 가 져갈 수 있는 재화도 극히 한정되 있었다. 그 결과, 상당수의 일본인 들이 공식귀환절차를 거치지 않고 부산항 부근에서 밀항선을 이용해 고국으로 돌아가고자 했다. 밀항자들은 검역을 거치지 않고 본국에 돌아왔기 때문에 감염병 유입의 주요 원인으로 지목되었다.^54)^ 일본 인들이 본국으로 밀항을 해 돌아가는 현상을 두고 “경성일본인세화 회 (京城日本人世話会)”^55)^는 기관지를 통해 밀항은 “일본인 전체를 불행하게 하는 행위”라며 자제를 촉구했다.56

재조선 일본인들은 밀항을 위해 작은 규모의 어선이나 무역선을 주로 이용했는데, 바다를 건너던 중 태풍을 만나거나 기뢰로 인해 침몰하는 사고가 적지 않게 발생했다. 때로는 해적의 습격을 받기도 했는데, 이러한 위험에도 불구하고 일본인 밀항자들은 끊이지 않았 다.^57)^ 후쿠오카현청 사회과 직원의 증언에 따르면, 1945년 9월과 10 월 사이에 후쿠오카로 돌아온 귀환자들 중에는 공식 귀환선을 타고 돌아온 자들 보다 밀항으로 들어온 이들의 숫자가 더 많았다고 한 다.^58)^ 옛 식민지 관료들 중에서도 밀항을 택하는 자들이 있었는데, 그들은 더 많은 재산과 운반 금지품목을 일본으로 가져가기 위해 밀 항선에 올랐다. 예를 들어, 전 경성부 총무부장 바바 마사요시 (馬場 政義)와 동 경제과장 아베 세이이치 (安部誠一) 등은 밀항을 위해 조 선 목선을 55,000원에 구입했다. 그들은 1945년 10월 25일 오후 6시 경 한강 연안을 출발하려는 찰나에 발각되었는데, 선내에는 70명의 밀항자와 현금과 무기 등이 담긴 7백 개나 되는 수화물이 탑재해 있 었다.^59)^

허가 없이는 입항이 금지된 줄도 모르고 식민지시기처럼 자유롭게 조선을 드나들 수 있다고 생각해 부산항으로 들어왔다가 상륙금지를 당하거나 억류되는 일본인들도 적지 않았다. 이와 관련해 일본 외무 성의 외국 (外局)인 종전연락사무국이 SCAP에 문의한 결과, 일본인 은 현 시국에서 조선에 도항하는 것이 원칙적으로 불가능하다는 대 답이 돌아왔다. SCAP의 허가를 받았을 경우에 한해서 도항이 가능 했는데, 입항할 때 반드시 연합군 당국이 발행한 허가서를 지참해야 했다. 만약 SCAP의 허가 없이 조선으로 도항하는 일본인들이 계속 늘어난다면 일본인 전체를 대상으로 도항금지령이 내려질 가능성도 우려되는 상황이었다.^60)^

일본에서 밀항선을 타고 본국으로 돌아오는 조선인들도 적지 않았 다. 일본에서 본국으로 돌아가는 구 식민지 출신들이 지참할 수 있 는 현금은 최대 500엔 (円)까지 였고, 1945년 11월 25일 이후에 겨우 1,000 엔 (円) 으로 인상되었다.^61)^ 1,000 엔은 당시 담배 스무 갑 가 격에 해당하는 금액으로, 조선인들이 본국으로 귀환하기 위해서는 일본에서 차별과 과도한 노동을 견디면서 모은 재산의 대부분을 포 기해야 했다.^62)^ 이러한 이유로 본국으로 돌아오는 조선인 밀항자들 도 끊이지 않았는데, 1945년 9월 27일부터 11월 17일까지 약 48,000 명의 조선인이 밀항선을 타고 부산으로 들어왔다. 뿐만 아니라 12월 18일까지 마산으로 밀항해 온 조선인은 총 19,000명에 달했다.^63)^ 조 선인 밀항자 역시 검역을 거치지 않은 상태라 공중위생의 큰 위협으 로 간주되었다. 따라서 밀입국하다 발각된 조선인들은 부산항에 설 치된 집합소로 보내져, 그곳에서 건강상태를 확인한 후 천연두와 장 티푸스 예방 접종을 받게 되었다.^64)^ 조선과 일본을 오가는 밀항선을 단속하기 위해, 부산항에서는 야간 구축함이 15분 마다 탐조등을 밝 히며 감시를 늦추지 않았다.^65)^

1946년 봄부터 밀항선을 타고 일본으로 향하는 조선인들도 증가하 기 시작했다. 이 시기 일본으로 들어간 조선인 밀항자들은 모두 전 후 일본에서 조선으로 돌아온 귀환자들이었는데, 그들 중 약 80%는 생활고로 인해 다시 일본행을 택한 것이었다.^66)^ 36년간 이어진 일제 의 식민지배로 인해 조선사회의 곳곳에는 빈곤과 혼란이 만연했고, 해방의 기쁨을 맛보기도 전에 국토는 외세에 의해 분단되었다. 이러 한 상황에서 고국으로 돌아온 귀환자들은 제대로 된 일거리를 구하 기가 어려웠다. 조선에 돌아올 때 현금을 1인당 1,000엔까지 밖에 지 참할 수 없었기 때문에, 귀환자들은 집과 식량을 구하는 것도 힘든 상황이었다. 주요 입항지인 부산의 경우 상황은 더욱 심각했다. 1946 년 2월까지 부산으로 들어온 귀환자 중에 약 20만 명이 갈 곳이 없 어 항구 주변에 잔류했다. 그 결과 부산항 주변의 인구가 급증하면 서 주택난과 식량 궁핍, 물가폭등, 실업 등 사회문제가 불거지고 불 법 외환거래와 대일 밀수출 등이 더욱 기승을 부리게 되었다. 또한 열악한 위생환경으로 인해 각종 질병까지 창궐하는 상태였다.^67)^ 결 국 생활고를 견디지 못하고 부산항 부근에서 밀항선을 구해 다시 일 본으로 돌아가려는 자들이 하나 둘씩 생겨난 것이다. 7월에만 밀항 을 하다 일본영해에서 체포된 조선인이 7,378명에 달했는데, 밀항에 성공한 이들의 숫자를 고려하면 한 달에 약 1만 명의 조선인이 일본 으로 들어왔다고 추산되었다.^68)^

콜레라 유행으로 인한 식량난도 귀환 조선인들을 밀항으로 내 모 는 이유가 되었다. 보건당국은 콜레라가 확산되는 것을 막기 위해 환자 발생지역을 중심으로 교통을 차단하는 봉쇄정책을 단행했는데, 이로 인해 물자공급에 차질이 생겨 식량부족현상이 더욱 심각해졌 다. 교통차단의 첫 번째 대상이 된 부산 초량의 경우, 주민들은 감시 가 소홀한 밤이나 새벽을 이용하여 식량을 구하기 위해 타 지역으로 넘어가기도 했다. 그 결과 콜레라가 외부로 퍼져 나갈 위험이 더욱 커졌다.^69)^ 더욱이 6월에는 20년 만에 가장 심각한 수해가 발생하여 약 20%의 농작물이 유실되기도 했다.^70)^ 식량난이 갈수록 심각해지 자, 7월에는 2천여명의 군중들이 부산부청에 모여 시위를 하는 쌀소 동이 일어났다. 화난 이들은 부청 건물의 유리 20여 장을 깼는데, 그 과정에서 이를 저지하려던 문서계장이 부상을 입기도 했다. 사태의 중대성에 놀란 당국은 소방차를 출동시켜 군중들을 해산시키려 했으 나 분노한 군중들은 좀처럼 물러서지 않았다. 결국 당국이 식량배급 을 약속하고 동시에 미군전차가 출동을 하면서 군중은 해산했다.^71)^ 다른 지역에서도 상황은 크게 다르지 않았는데, 특히 생활고를 겪고 있던 귀환자들의 사정은 더욱 비참했다. 합천의 경우, 문전걸식으로 겨우 생명을 이어오던 귀환자 백 수십명이 교통차단으로 더 이상 구 호를 받을 수 없어 아사상태에 빠지기도 했다.^72)^ 울산에서도 배고픔 을 견디지 못해 방어진항에서 밀항선을 타고 일본으로 건너가려는 귀환자들이 끊이지 않았다. 이에 밀항 단속에 그치는 것이 아니라 그들의 생활을 보장해 줄 행정당국의 근본대책이 필요하다는 지적도 나왔다.^73)^ 조선인 밀항자를 두고 ‘일본이 그리 좋아서 돌아가느냐’고 빈정대는 이들도 있었지만, 위험을 무릅쓰고 다시 일본으로 돌아갈 수밖에 없는 상황을 식자층은 심각하게 받아들였다. 또한 밀항이 근 절되지 못하는 상황에 제대로 대처하지 않는 당국의 소극적인 태도 도 비판되었다.^74)^

이처럼 조선인 밀항자들이 눈에 띄게 증가하자, 미군정청은 밀항 자에 대한 단속을 한층 강화했다. 우선 5월 15일, 외무처는 미군정청 허가 없이 영해나 영공 이외의 지역으로 불법 여행을 하거나 재산을 운반하는 행위 등을 금지한다는 취지의 법령 72호를 발표했다. 이 법령에 따르면 군정청 허가 없이 영해 이외의 지역에 선박 혹은 항 공기를 이동시키는 것 역시 법령위반으로 처벌의 대상이 되었다. 이 법령이 발표되기 전 까지 10일 간, 수 백여명의 조선인 밀항자가 일 본의 센자키와 시모노세키 (下関) 사이의 해안에서 체포되었고, 이에 사용된 선박은 모두 몰수되었다.^75)^

한 편, SCAP과 일본사회는 조선인 밀항자들을 콜레라 전파의 원 인으로 지목하며 단속을 강화하기 시작했다. SCAP은 1946년 6월 12 일 “일본으로의 불법입국 억제에 관한 각서”(SCAPIN 1015)를 통해 서 불법 입항선박 수색 및 체포를 명했다. 각서에는 “조선에서 불법 선박을 통해 들어오는 이들로 인해 일본으로 콜레라가 유입될 수 있 는 심각한 위험을 고려하여, 불법적으로 일본의 항구에 들어오는 선 박을 탐지하고 체포하기 위한 적극적인 조치를 취할 것”이 명시되어 있다.^76)^ 7월부터는 영국함대도 일본영해를 순찰하며 대일 밀항자를 단속하기 시작했다^77)^. 일본의 정치계도 조선인 밀항자로 인해 콜레 라가 일본으로 전파될 위험에 대한 우려의 목소리를 높였다. 1946년 8월 17일에 열린 제90회 제국회의 중의원 본회의에서 시이쿠마 사부 로 (椎熊三郎)는 “밀항단속 및 치안유지에 관한 긴급 질문(密航取締 竝に治安維持に關する緊急質問)”을 통해서 경찰권의 약화로 인해 ‘일본사회의 질서와 법규를 무시하는 조선인과 대만인’에 대한 단속 이 제대로 실시되지 못한다는 점을 지적했다. 이어, 한 때 본국으로 돌아간 조선인들이 밀항하여 다시 일본으로 들어오고 있는데, 그들 중에 콜레라나 장티푸스, 이질과 같은 감염병의 보균자가 많아 일본 인들 사이에서도 감염자들이 속출하고 있다고 강조했다.^78)^ 실제, 1946년 일본으로 밀항을 하다 체포된 조선인들 사이에서 콜레라를 비롯한 감염병 환자와 보균자가 다수 발견되었다 (표 1). 센자키항에 서 밀항 단속을 하던 뉴질랜드 군에 따르면 체포된 밀항선에서 콜레 라로 사망한 것으로 추정되는 아이들의 시체 여러 구가 발견되기도 했다.^79)^

그러나 최덕효와 조경희의 지적처럼 일본 정계에서 재일조선인과 조선인 밀항자 문제를 치안과 위생의 문제로 연결시키는 것은, SCAP의 점령 통치하에 진행된 ‘과도한 민주화’에 따른 경찰권 약화 를 문제 삼기 위한 의도가 포함되어 있다. 즉, 약화된 경찰권을 회복 시키고 재일조선인 사회에 대한 제재와 단속도 강화해 나가는 것을 목적으로 하고 있다.^80)^ 그리고 박사라의 지적처럼 콜레라 유입를 막 기위해 조선인 밀항자 단속을 명한 SCAP의 각서나 일본 정계의 움 직임은 재일조선인을 포함해 구 식민지출신을 대상으로 한 “외국인 등록령 (1947년 5월 2일)”을 발포하기까지의 과정에서 중요한 모멘 텀으로 작용했다. 이 법령으로 구 식민지 출신들은 ‘당분간 외국인으 로 간주되며’ 공식적으로 관리와 감시의 대상이 되었다.^81)^

역사적으로 감염병의 통제는 종종 질병 그 자체의 위험을 훨씬 넘 어선 과도한 방역을 수반하기도 한다. 특히 한 국가가 선택하는 방 역 전략은 생물학적 측면만큼 정치적인 의도가 반영되는 경우가 많 은데, 엄격한 국경 통제와 외국인 관리를 강화하는 명분으로 작용하 기도 한다.^82)^ 이러한 맥락과 유사하게, 일본은 콜레라가 자국에 먼저 유입되었고 이미 국내 유행으로 번진 상황에서 조선인 밀항자를 일 본 내 콜레라 전파의 원인처럼 다루며 재일조선인 사회 전체에 대한 제재의 논리로 발전시켰다. 게다가 상기한 바와 같이, 귀환선을 통해 콜레라가 일본으로 처음 유입 된 이유 중 하나가 출항지에서 검역을 담당하던 미군의 부주의 때문이었다. 또한 부산항에 콜레라환자를 실은 귀환선이 들어왔을 때에는, 콜레라 검역에 만전을 기하라는 권 고에도 불구하고 미군의가 콜레라를 의심하는 조선인 당국자를 불신 해 방역에 차질을 빚기도 했다. 그러나 미군의 방역 실패에 대한 SCAP의 반성적 태도는 찾아볼 수 없다. 궁극적으로 콜레라가 귀환 선을 통해 일본과 해방조선에 전파된 배경이 일본의 제국주의 팽창 과 침략전쟁이었다는 사실에 대한 문제제기도 생략되었다

더욱이 일본과 SCAP이 조선의 밀항자를 콜레라 전파의 원인으로 간주하는 태도는 그들 내부의 뿌리 깊은 차별정서와 감염병학적 오 리엔탈리즘에서 비롯된 것이라 할 수 있다. 상기한 바와 같이 미국 과 일본은 아시아를 질병의 온상으로 바라보았다. 그리고 피식민자 들의 높은 질병 감수성을 두고 그들이 처한 열악한 생활환경이나 만 성적 빈곤은 외면한 채, 그들을 ‘움직이는 감염체 보유 숙주 (mobile reservoirs)’로 간주하며 배제와 차별정책을 정당화했다.^83)^ 그 연장선 상에서 일본은 천연두나 발진티푸스 등이 자국내에 유행할 때 감염 병이 쉽게 전파되는 사회적 조건을 직시하기 보다 조선인이나 다른 피차별계층에게 그 발생 원인을 돌리기도 했다.^84)^ 이러한 태도는 일 찍이 부산 개항 직후 조선에 들어온 일본인 관리들 사이에서도 나타 났다. 1879년 나가사키 (長崎)에서 부산항으로 콜레라가 유입되었을 때, 당시 부산항에 주재한 관리관 마에다 켄키치 (前田献吉)는 테라 지마 무네노리 (寺島宗則) 외무경에게 서신을 보내 당시의 상황을 보고했다. 그는 ‘평소 불결하기 짝이 없는 조선인 사회에 콜레라가 퍼지고 있으므로 거류민 보호를 위해 조선인의 거류지 출입금지가 필요하지만 상법상 불가능하다’고 토로했다. 또한 마에다는 그 해 콜 레라가 일본에서 부산항으로 전파된 사실을 인지하면서도, 콜레라가 전라도지역의 풍토병일 가능성이 있다는 의견도 덧붙였다.^85)^ 67년이 지난 1946년에도 일본은 콜레라가 전파된 역사적 배경과 사회적 조 건은 생략한 채, 조선인 밀항자를 콜레라 전파의 원인으로 지목하며 조선인들에 대한 차별과 배제의 심리를 노골화해 갔다.

## v 맺는 말

일본의 패전이 선언되자 구 (舊) 일본제국 내에서는 전례 없는 규 모의 귀환행렬이 시작되었다. 당시 일본은 패전에 따른 심리적 허탈 감과 빈곤에 허덕이고 있었다. 게다가 열악한 보건위생 상태에 식량 부족까지 겹치면서 사람들은 각종 감염병에 노출되었다. 일제의 오 랜 수탈과 전쟁동원의 대상이 되었던 조선을 비롯해 일본의 옛 식민 지의 사정은 훨씬 나빴다. 이러한 상황에서 수 백만에 달하는 귀환 자들의 이동은 감염병이 전파되는데 최적의 조건이 되었다. 여러 감 염병 중 특히 우려되었던 것은 콜레라였다. 콜레라는 수인성 감염병 으로 콜레라균에 오염된 음식물을 섭취하거나, 드물지만 콜레라 환 자나 보균자의 구토와 배설물을 직접 접촉한 경우 감염되기도 한다. 다시 말해, 짧은 기간 동안 전례 없는 규모의 귀환자들이 좁고 비위 생적인 귀환선을 타고 본국으로 돌아오는 과정은 콜레라가 각지로 전파되기 쉬운 환경을 형성했다. 전후 소련과 대치하며 극동지역에 서 새로운 지정학적 질서를 세우려고 하는 미국에 있어서 38선 이남 의 조선과 일본사회의 안정은 무엇보다 중요했다. 따라서, 권역 내의 질서 유지는 물론 주둔군의 안전을 위해 미군은 입출항 검역을 포함 한 공식적인 귀환작업을 실시했다.

일반적으로 검역은 개인에 대한 국가권력의 표출이자 한 나라의 통치권을 발휘하는 수단으로 활용되기도 한다.^86)^ 이러한 점을 미군 의 감독하에 부산항과 일본에서 실시한 검역에 비추어 볼 때, 귀환 자 검역은 고국으로 돌아오는 이들에게서 ‘병원성 요소들’을 제거하 는 역할도 했지만 동시에 그들이 처한 상황을 각인시키는 과정이기 도 했다. 즉, 검역절차를 거치면서 일본인들은 그들이 이제 패전국의 국민이며 미군의 통치대상이라는 점을 인식하게 되었다. 어렵게 고 국으로 돌아온 조선인들 역시 본국에서 처음 마주한 현실은 미군이 지휘하는 DDT 살포였다. 다시 말해, 귀환자 검역은 일본인과 조선인 들이 새로운 통치 질서에 들어서는 첫 관문이기도 했다. 그러한 점 에서 밀항은 보건위생에 위협이 되는 동시에 미군의 통치 질서를 훼 손하는 행위로 간주되었다.^87)^ 특히 1946년 봄부터 일본과 조선에 콜 레라가 유행하면서, 일본으로 향한 조선인 밀항자 문제가 더욱 부각 되었다. SCAP은 조선인 밀항자들로 인해 일본으로 콜레라가 유입되 는 것을 막기 위하여 해안 경계를 강화했다. 일본 정계 역시 조선인 밀항자를 콜레라 전파의 주범이자 사회질서를 위협하는 존재로 묘사 하며 강력한 단속을 촉구했다. 이러한 움직임은 재일조선인 사회에 대한 차별과 배제를 공식화 해 나가는 과정과 맞닿아 있다.

문제는 콜레라가 중국에서 다른 아시아 지역으로 전파된 원인에 대 한 미군의 반성은 물론, 일본으로 콜레라가 유입되어 이미 유행하고 있다는 사실관계는 사라진 채 밀항 조선인을 콜레라 전파의 원인처럼 묘사하고 있다는 점이다. 이렇게 조선인 밀항자와 콜레라를 연결시키 는 논리의 기저에는, 미국과 일본사회에 뿌리 내린 감염병학적 오리 엔탈리즘이 존재한다고 할 수 있다. 즉, 감염병의 유행을 일으키는 사 회 내재적 요인들을 직시하기 보다, 그 원인을 타자 (아시아)에서 찾 으며 책임을 전가하는 것이다. 더욱이 전후 수 백만 명에 달하는 귀환 자들이 생겨난 역사적 배경은 물론, 고국으로 돌아간 조선인들이 생 활고로 인해 일본행 밀항선을 타게 된 근본적인 원인이 일본의 제국 주의 침탈과 전쟁이라고 하는 문제의 본질은 제거되었다. 다시 말해, 조선인 밀항자들로 인해 콜레라와 같은 위협적인 감염병의 침입에 노 출된 ‘피해자 일본’이라는 점 만을 부각시키고 있는 것이다. 이는 미 국이라는 강력한 제3자의 개입 하에서 다민족으로 구성되었던 제국의 과거를 지워버리고, ‘단일 민족국가’로 그 정체성을 재정립해 나가는 전후 일본사회의 단면을 축약적으로 나타낸다고 할 수 있다.

## Figures and Tables

**<그림 1>:**
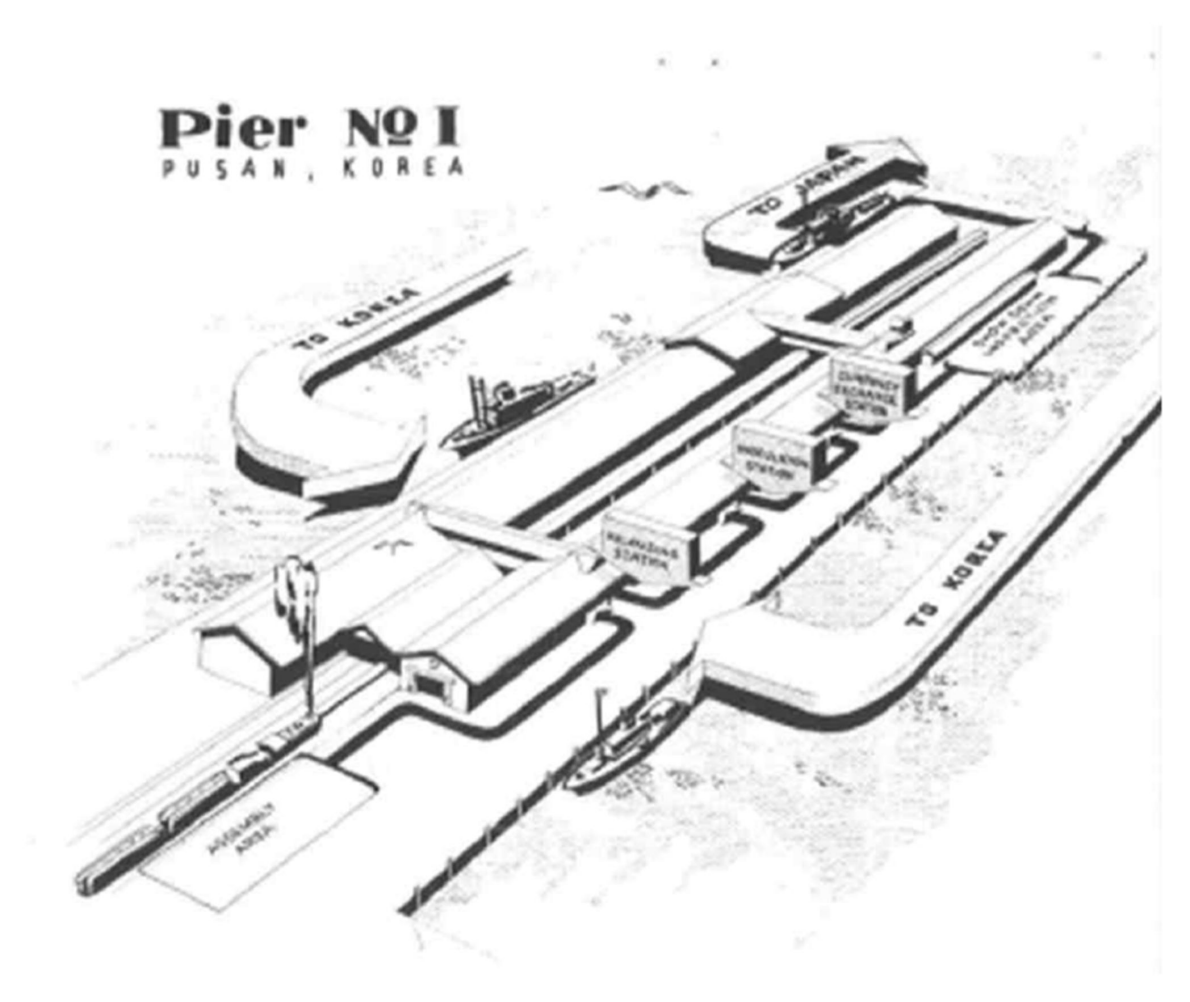
부산항 제1부두와 귀환작업 절차 출처: John M. Bullit (1945), 40th Infantry Division: History of Evacuation and Repatriation Through the Port of Pusan: Korea, 28 Sept. 45-15 Nov. 45, Place of publication not identified: publisher not identified.

**<사진 1>:**
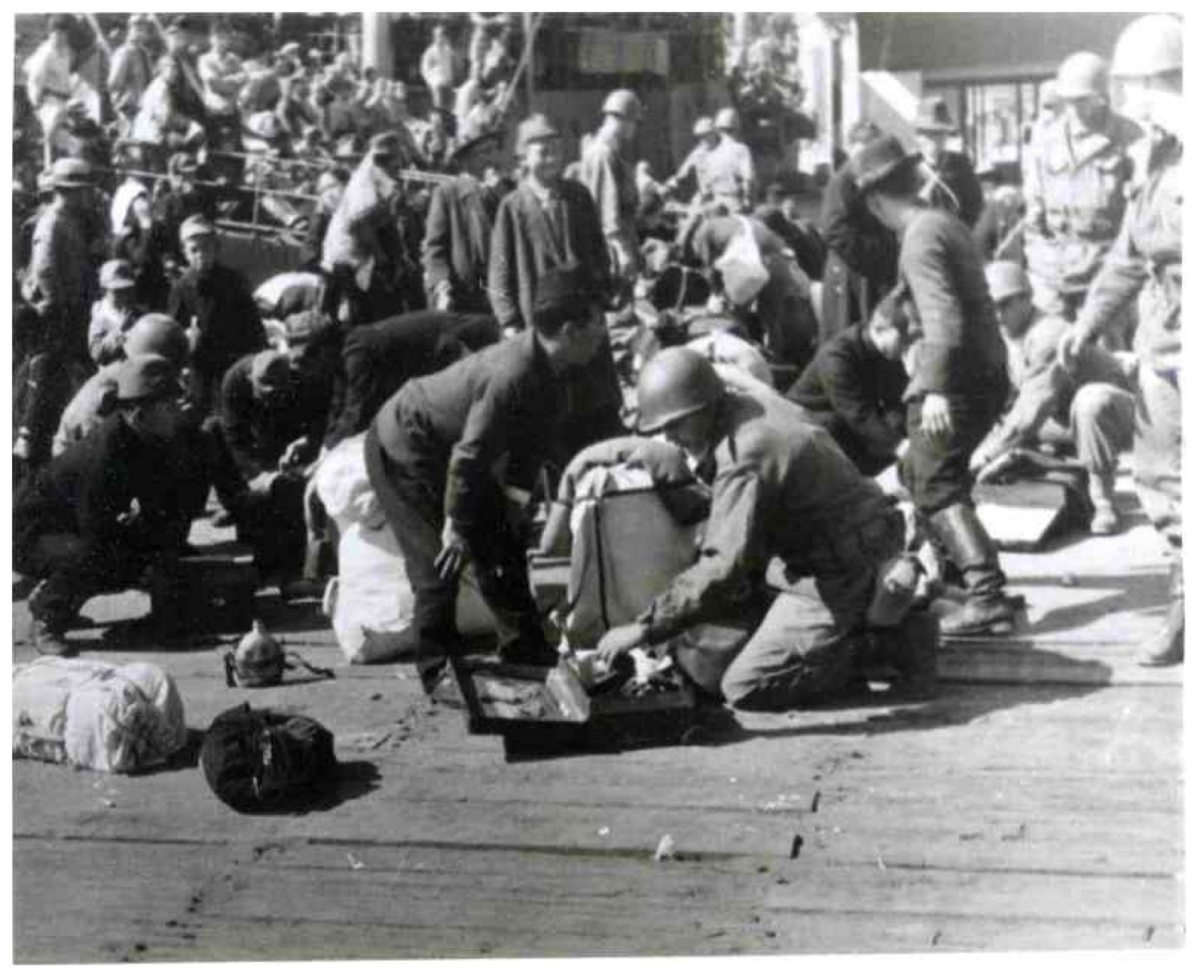
부산항 제1부두에서 일본인 귀환자의 짐을 수색중인 미군 출처: General Headquarters, Far East Command, Supreme Commander Allied Powers, and United Nations Command (1945-1948), “Searching outgoing Japanese repatriates at Pier No.1, Pusan, 24 Oct, 1945”, Photographs of Japanese Repatriation, RG 554 Records of General Headquarters, Far East Command, Supreme Commander Allied Powers, and United Nations Command, USAFIK: XXIV Corps, G-2 Historical Section, 국립중앙도서관 소장.

**표 1:** 1946년 밀항 조선인 대상 검역 결과

		센자키 (仙崎)	하카타 (博多）	사세보 (佐世保）	가라쓰 (唐津)	합계
천연두	환자	1	-	1	-	2
	사망자	-	-	-	-	-
콜레라	환자	31	1	150	2	184
	사망자	9	1	31	-	41
콜레라 보균자		51	-	162	-	213

출처: 山下喜明 (1975), “昭和20年代の検疫史”, 医学史研究, 44, pp.1-10

## Footnotes

1) 귀환자의 일본어 표현인 ‘히키아게샤 (引揚者, 인양자)’는 부두에서 물건 등을 실어 나르는 뜻의 히키아게루 (引揚げる)에 사람을 뜻하는 접미사 자 (者)를 결합시킨 단어이다. 이 단어는 패전 후 식민지/점령지 등에서 귀환 한 일본인을 지칭하는 것으로, 일본제국주의라는 역사적 배경 하에서 식민 자로 존재했던 그들의 정체성은 삭제된 채 ‘돌아온 자들’에 방점이 찍힌 표현이라고 할 수 있다 (Lori Watt (2009), *When Empire Comes Home: Repatriation and Reintegration in Postwar Japan,* Cambridge and London: Harvard University Press, pp.56-57 참조). 본 고는 일본인 귀환자를 포함한 구 (舊) 제국 일본 내에서 실시된 귀환작업을 연구대상으로 하기 때문에, 인양자가 아니라 포괄적인 의미의 귀환자 (歸還者, repatriate) 를 사용한다.

2) 위의 책 Lori Watt (2009), *When Empire Comes Home*, p.2; Araragi Shinzō (translated by Sherzod Muminov) (2017), “The Collapse of the Japanese empire and the great migrations: repatriation, assimilation, and remaining behind”, In Barak Kushner and Sherzod Muminov (eds), *The Dismantling of Japan’s Empire in East Asia: Deimperialization, postwar legitimation and imperial afterlife*, London: Routledge, pp.66-83.

3) John Dower (1999), *Embracing Defeat: Japan in the Aftermath of World War* *II*, London: Penguin, p.89.

4) 콜레라는 콜레라균 (Vibrio cholerae) 에 의해 발생하는 수인성 급성 감염병이 다. 콜레라에 감염된 환자는 구토와 발열, 복통을 앓고 극심한 설사로 탈수 현상을 겪게 된다. 치료를 받지 못한 환자는 몇 시간 내에 사망에 이르기도 한다 (“Cholera”, WHO, https://www.who.int/news-room/fact-sheets/detail/cholera).

5) 조경희 (2013), “전후 일본 ‘대중’의 안과 밖 -암시장 담론과 재일조선인의 생활세계-”, 현대문학연구, 50-0, 141-178쪽.

6) Deokhyo Choi (2021), “The Empire Strikes Back from Within: Colonial Liberation and the Korean Minority Question at the Birth of Postwar Japan, 1945 - 47”, *The American Historical Review*, https://doi.org/10.1093/ahr/rhab199.

7) Tessa Morris-Suzuki (2010), *Borderline Japan: Foreigners and Frontier Controls in the Postwar Era*, Cambridge: Cambridge University Press.

8) 김정란 (2021), “제국의 흔적 지우기: 패전 후 일본에서의 귀환자 검역”, 최 해별 편, 질병 관리의 사회문화사: 일상생활에서 국가정책까지, 서울, 이 화여자대학교출판문화원, 291-321쪽.

9) 위의 책 Lori Watt (2009), *When Empire Comes Home*, p.190; 아사노 도요미 (2011), “사람, 물자, 감정의 지역적 재편과 국민적 학지 (學知)의 탄생: 절 첩 (節疊)된 제국의 재산과 생명의 행방”, 한림일본학, 19-0, 85-118쪽.

10) Allied Geographical Section SWAP, *Special Report No.111 Pusan Korea*, 31 Aug 1945, WO 252/999, The National Archives.

11) Jeong-Ran Kim (2013), “The Borderline of “Empire”: Japanese Maritime Quarantine in Busan c.1876-1910”, *Medical History*, 57-2, pp.226-248.

12) 조선에 주둔하던 일본육군 중에는 진해와 인천, 제주를 떠나 일본으로 돌아간 경우도 있었고, 군산이나 목포, 마산, 제주 등을 통해 귀국한 조 선인들도 존재했다 (General Headquarters, Far East Command, Supreme Commander Allied Powers, and United Nations Command (1945-1948), “Movement of Displaced Persons Since 28 Sept 1945”, *Japanese Repatriation*, RG 554 Records of General Headquarters, Far East Command, Supreme Commander Allied Powers, and United Nations, USAFIK: XXIV Corps, G-2 Historical Section, 1945-1948, 국립중앙도서관)

13) Crawford F. Sams (1988), *“Medic”: The Mission of an American Military Doctor in Occupied Japan and Wartorn Korea*, Armonk, N.Y. and London: M.E. Sharpe, pp.89-91.

15) “Joint Army-Navy Intelligence Study” 연속 보고서는 미군 합동참모본부의 합동 정보 위원회가 설립한 합동 정보 연구 출판 위원회에서 1943년 4월 부터 1947년 7월까지 발행한 것이다. 이 연속 보고서는 미군이 제2차 세 계대전을 치르면서 특정지역에서의 군사작전과 점령준비에 필요한 정보 를 수집하고 활용하기 위한 목적으로 제작되었다. 1944년 봄부터 미군의 주된 관심은 일본과 그 식민지로 옮겨갔다 (Medical Department, United States Army (1955), *Preventive Medicine in World War II, Vol3 Personal Health Measures and Immunization*. Washington, D.C.: The Surgeon General Department of the Army, p.270).

16) Joint Intelligence Study Publishing Board (1944), *Joint Army-Navy Intelligence Study of Southwest Japan: Kyūshū, Shikoku, and Southwestern Honshū*, Washington: Joint Intelligence Study Publishing Board.

17) Joint Intelligence Study Publishing Board (1945), *Joint Army-Navy Intelligence Study of Korea, Including Tsushima and Quelpart*, Washington: Joint Intelligence Study Publishing Board.

18) John M. Bullit (1945), *40^th^ Infantry Division: History of Evacuation and Repatriation Through the Port of Pusan: Korea, 28 Sept. 45-15 Nov. 45*, Place of publication not identified: publisher not identified, p.29.

19) John Dower (1999), *Embracing Defeat*, pp.72-74.

20) John M. Bullit (1945), *40^th^ Infantry Division*, p.29.

21) *Ibid*., p.19.

22) SCAP (1945), *Summation of Non-Military Activities in Japan and Korea*, No.2, Tokyo: General Headquarters, Supreme Commander for the Allied Powers; General Headquarters, Far East Command, Supreme Commander Allied Powers, and United Nations Command (1945-1948), *Repatriation Information Book, September 25 - December 31 1945*, RG 554 Records of General Headquarters, Far East Command, Supreme Commander Allied Powers, and United Nations Command, USAFIK: XXIV Corps, G-2 Historical Section, 국 립중앙도서관 소장, pp.29-32.

23) 溝口元(1999), “公衆衛生―引揚検疫とDDT”, 中山茂編, 通史日本の科学 技術１ 占領期, 東京: 学友書房, pp.251-259.

24) SCAP (1945), *Summation of Non-Military Activities in Japan and Korea*, No.2.

25) 厚生省(1949), 衛生行政業務報告：厚生省報告例　昭和21年, 東京: 厚生省 大臣官房統計情報部, pp.1-14; 厚生省公衆衛生編(1980), 検疫制度百年史, 東京: ぎょうせい,

26) Public Health and Welfare Section of GHQ/SCAP (1946-51), “Public Health and Welfare in Japan”, *The name of Series: Reparations and Public Welfare Activities, 1946-51*, Tokyo: PHW, pp.68-69.

27) 김정란 (2021), “제국의 흔적 지우기”.

28) 引揚援護庁(1950), 引揚援護の記録, 東京: 引揚援護庁, p.77.

29) Robert John Perrins (2009), “For god, Emperor and Science: Competing Visions of the Hospital in Manchuria, 1885-1931” in Mark Harrison, Margaret Jones and Helen Sweet (eds) *From Western Medicine to Global Medicine: The Hospital Beyond the West*, New Delhi: Orient Blackswan Private Limited, pp.67-107.

30) 釜山府(1937), ‘釜山ニ管理官及ビ医員ヲ置ク議’, 釜山府史原稿5, 釜山：釜 山府, pp.161-162.

31) 위의 책 Lori Watt (2009), *When Empire Comes Home*, pp.15, 92; Christopher Aldous and Akihito Suzuki (2012), *Reforming Public Health in Occupied Japan, 1945-52: Alien prescriptions?*, Abingdon: Routledge, pp.90-91.

32) PHW (1946-51), “Public Health and Welfare in Japan”, pp.68-69; MM Crawford F. Sams (1988), *“Medic”*, pp.89-91.

33) 厚生省(1946), *“引揚船中ニ多発セルコレラ流行ニ関スル状況報告”, 幣原 内閣次官会議書類*, April 18, Ref: A17110921000, https://www.jacar.archives.go.jp/aj/meta/MetSearch.cgi, Access: March 15, 2021.

34) General Headquarters, Far East Command, Supreme Commander Allied Powers, and United Nations Command (1945-1948), “Prevention and Control of Cholera”, 1945.11.15, *Public Health and Welfare of Troops*, RG 554 Records of General Headquarters, Far East Command, Supreme Commander Allied Powers, and United Nations Command, USAFIK: XXIV Corps, G-2 Historical Section, 국립중앙도서관 소장.

35) 위의 책 Crawford F. Sams (1988), *“Medic”*, pp.89-91.

36) 山下喜明(1975), “昭和20年代の検疫史”, 医学史研究, 44, pp.1-10; 山本俊 一(1982), 日本コレラ史, 東京：東京大学出版会, pp.206-209.

37) 厚生省(1949), *衛生行政業務報*, pp.1-13.

38) 厚生省公衆衛生編(1980), 検疫制度百年史, pp.76-77; 児玉威(1947), “戦中 戦後の防疫”, 日本医事新報, 1230, pp.6-10.

39) GHQ/SCAP, “Quarantine Procedures for Cholera in Repatriates”, *Public Health and Welfare of Troops*.

40) “三千名ㆍ死의 恐怖, 海上에서 陸地同胞에 嘆願虎疫發生으로 歸還船接陸 禁止”, 부산신문, 1946.5.5.

41) 厚生省公衆衛生編(1980), 検疫制度百年史, pp.78-79; 山本俊一(1982), 日本 コレラ史, pp.206-208.

42) “釜山港에 虎列刺! 中支同胞歸還船中에서 患者發生”, 중앙신문, 1946.5.7.

43) “防疫義勇隊組織”, 민주중보, 1946.5.9; “不安의 虎列刺船은? 患者는 病院 船에 隔離”, 민주중보, 1946.5.16.

44) “부산에 호열자침입, 본도당국서 방역완벽기해 활동중”, 영남신문, 1946.5.9.

45) “虎疫菌은 發見안됏다 美軍醫가 方今歸還船調査中”, 부산신문, 1946.5.17.

46) “호열자 遂상륙? 진성으로 인정 방역대책”, 부산신문, 1946.5.23.

47) “영도필담”, 부산신문, 1946.5.23.

48) “귀환동포선에서 호역 침입 금후 각지로 파급의 위험 부산, 대전, 인천서 44명 발견”, 자유신문, 1946.5.23.

49) “四十四名中卄七名이 眞性, 釜山虎列刺患者發生狀況”, “귀환선에 검역, 격 리병실도 시설”, 중앙신문, 1946.5.26.

50) “남한 콜레라환자가 1335명에 달함”, 동아일보, 1946.6.23.

51) SCAP (1945-1948), “INFO: CG KBC”, 1946.7.13, *Japanese Repatriation*.

52) 위의 책 Crawford F. Sams (1988), *“Medic”*, pp.89-91, pp.217-218.

53) “釜山通信”, 京城内地人世話会会報, 1945.10.4.

54) General Headquarters, Far East Command, Supreme Commander Allied Powers, and United Nations Command (1945-1948), Interview with 1^st^ Lt W. J. Game, DP Office, Foreign Affairs, 1945.10.15, *Repatriation & Removal of Peoples in Pusan Areas*, RG 554 Records of General Headquarters, Far East Command, Supreme Commander Allied Powers, and United Nations Command, USAFIK: XXIV Corps, G-2 Historical Section.

55) 일본인세화회는 ‘재조일본인의 보호’를 목적으로 한 민간단체로, 1945년 8 월18일에 조선에 거주하던 유력 재계인, 언론인, 대학관계자 등이 중심이 되어 ‘거류민 생활의 원호, 귀환 관리, 미군과의 교섭’ 등을 위해 경성일본 인세화회가 발족한 것이 그 시초이다. 이후 해방 조선 내의 주요 도시에 도 일본인세화회 지부가 결성되었다 (森田芳夫(1953), 秘録大東亜戦史朝 鮮篇朝鮮引揚史, 東京: 富士書苑, p.38).

57) GHQ/SCAP (1945-1948), “Processing and Evacuating”, *Repatriation & Removal of Peoples in Pusan Areas;* G-2 (1945-1948), “The 40^th^ Division Occupies Southeastern Korea”, *History of United States Army Forces in Korea, Part 1*, http://db.history.go.kr/item/level.do?itemId=husa, access 12 August, 2018.

58) 西日本図書館コンサルタント協会編(1980),ある戦後史の序章：MRU引揚医 療の記録, 福岡：西日本図書館コンサルタント協会, pp.13-14.

59) “거금과 무기를 滿載한 일본인 70명 밀항단”, 자유신문, 1945.10.27.

60) “日本人の南鮮渡航について”, 京城内地人世話会会報, 60, 1945.11.14.

61) GHQ/SCAP (1945-1948), *Repatriation Information Book, September 25 - December 31 1945*, p.69.

62) 위의 책 Tessa Morris-Suzuki (2010), *Borderline Japan*, pp.65-66.

63) GHQ/SCAP (1945-1948), *Repatriation Information Book, September 25 - December 31 1945*, pp.49-51.

64) *Ibid*., 70.

65) GHQ/SCAP (1945-1948) “Japanese Civilians at Pusan, Korea”, 1945.10. 24-25, *Repatriation & Removal of Peoples in Pusan Areas*.

66) 위의 책 Tessa Morris-Suzuki (2010), *Borderline Japan*, p.67.

67) “전재 귀환동포 20만, 부산잔류”, 조선일보, 1946.2.11.

68) “일본에 밀항 격증, 7월중에 만명이라고”, 부산신문, 1946.08.20.

69) “怪疾遮斷區에 쌀難對策업시는 鐵壁은 虛言”, 민주중보, 1946.5.29.

70) “맥아더 6월 정례보고서에 조선의 수재와 콜레라만연 보고”, 동아일보, 1946.8.27.

71) “마침네 港都의 饑餓聲爆發! 一縷의 糧道求한 高喊! 爲政者는 무엇하나”, 민주중보, 1946.7.7.

72) “遮斷苦에우는 同胞”, 민주중보, 1946.8.10.

73) “渡日群이 雲集?”, 민주중보, 1946.8.4

74) “굶주림에 일본으로 밀항하는 동포들”, 국민일보, 1946.8.21.

75) “일본 밀항은 군정 위반, 수백 명의 조선인을 체포”, 자유신문, 1946.5.16; “외무처, 영해나 영공 이외 불법여행을 금지”, 동아일보, 1946.5.16.

76) SCAP (1946), “Suppression of Illegal Entry into Japan (SCAPIN 1015)”, *Supreme Commander for the Allied Powers Directives to the Japanese Government* (SCAPINs), 1946.6.12.

77) “대일밀항자주의, 영국함대가 출동경계”, 중앙신문, 1946.7.20.

78) “密航取締竝に治安維持に關する緊急質問（椎熊三郎君提出)”, 第90回帝国 議会衆議院本会議第30号昭和21年8月17日, URL : https://teikokugikai-i.ndl.go.jp/simple/detail?minId=009013242X03019460817&spkNum=11.

79) 위의 책 Tessa Morris-Suzuki (2010), *Borderline Japan*, p.56

80) 조경희(2013), “전후 일본 ‘대중’의 안과 밖”; Deokhyo Choi (2021), “The Empire Strikes Back from Within”.

81) 朴沙羅(2013), “ お前は誰だ！ ―占領期における朝鮮人と不法入国の 定義をめぐって―”, 社会学評論, 64-2, pp.275-293.

82) Alison Bashford (2016), “Maritime Quarantine: Linking Old World and New World Histories” in Alison Bashford (ed) *Quarantine: Local & Global Histories*, London: Palgrave, pp.1-12; 김정란 (2020), “불안과 불확실성의 시대: 19세 기 콜레라 대유행과 21세기 코로나19 팬데믹”, 해양유산, 2, 9-35쪽.

83) 顧雅文(2005), “植民地期台湾における開発とマラリアの流行：作られた悪 環境”, 社会経済史学, 70-5, pp.583-605; Jeong-Ran Kim (2016), “Malaria and Colonialism in Korea, c.1876-c.1945”, *Social History of Medicine*, 29, pp.360-383.

84) Aldous and Akihito (2012), *Reforming Public Health in Occupied Japan, 1945-52*, pp.90-91.

85) 外務省(1949), ‘肝心コレラ病ニ関スルノ景況申上ノ件’ 1879.8.5, 日本外交 文書第十二巻, 東京：日本国際連合協会, pp.230-233.

86) Mark Harrison (2016), “Afterword”, in Alison Bashford (ed), *Quarantine*, pp.251-257.

87) Jeong-Ran Kim, “Surveillance, Policing and Filtration: Quarantine against Repatriates in Busan after WWII”, in Takeshi Onimaru (ed.) *(Dream of) Prevention and Control: Public Health, Policing, and Colonial State in Asia*, Joint NUS/Kyoto Press (forthcoming).
